# Nosological Characteristics in Women with Social Media Disorder: The Role of Social Functional Impairment and Agreeableness

**DOI:** 10.3390/ijerph192215016

**Published:** 2022-11-15

**Authors:** Lara Scherer, Lisa Mader, Klaus Wölfling, Manfred E. Beutel, Boris Egloff, Kai W. Müller

**Affiliations:** 1 Outpatient Clinic for Behavioural Addictions, Department of Psychosomatic Medicine and Psychotherapy, University Medical Centre of the Johannes Gutenberg University Mainz, 55131 Mainz, Germany; 2 Department of Psychosomatic Medicine and Psychotherapy, University Medical Centre of the Johannes Gutenberg University Mainz, 55131 Mainz, Germany; 3 Department of Psychology, Personality Psychology and Psychological Assessment, Johannes Gutenberg University Mainz, 55122 Mainz, Germany

**Keywords:** social media disorder, internet-related disorder, gender, personality, comorbidity

## Abstract

Social media disorder (SMD) is a frequently occurring subtype of Internet-related disorders (IRD), which has recently become a focus of research. It is noticeable that women are among those affected, whose nosological characteristics need to be examined. A clinical sample of *n* = 294 women (14–68 years, M = 36.88 years) was generated. The questionnaire included questions about demography, IRD, SMD, personality traits, psychopathological distress, functional impairment and comorbid mental illnesses. IRD was found in 17.5 percent and SMD in 12.5 percent of women. Compared to women with global IRD Women with SMD reported lower scores on the personality traits neuroticism and agreeableness. They are more frequently functionally impaired in the social dimension, more often reported comorbid substance-dependency and less eating disorders. The results suggest that although have similar characteristics to the comparison group, women with SMD differ in their nosological characteristics from women with global IRD.

## 1. Introduction

Internet-related disorders (IRD), clinically relevant health issues, have been researched for over two decades. Research is characterized by either focusing on IRD as a global construct or addressing specific subtypes of IRD, especially (Internet) Gaming Disorder. 

The distinction of specific subtypes under the umbrella of IRD is of importance since research has indicated different nosological characteristics within specific subtypes [1,2,3]. Nosology is the pathology, the systematic description of a disorder or disease. In this case, we try to identify specific characteristics (inter alia personality traits, comorbidities, psychopathological distress, etc.) that distinguish SMD from other subtypes of INS. In particular, Internet Gaming Disorder (IGD) has been examined in detail in recent years. As a result, it was included in DSM-5 [4] in 2013 and recognized as an official disorder in the forthcoming ICD-11 [5]. Other subtypes will be coded as “other specific forms of dependent behavior” [6].

Research on the addictive use of social media or social networking sites (Social Media Disorder, SDM; labeled by Van den Eijnden and colleagues [7]) came increasingly to the fore. Social media enable consumers to create, share and evaluate content (e.g., web blogs, Wikipedia, Instagram, Youtube), while social networking sites offer the opportunity to join larger networks with a focus on interactive components (e.g., Linked In, Facebook, Whatsapp). Both terms share social interaction on the Internet and yet they cover different applications [8], although more and more platforms connect both areas. Social media disorder includes applications of both categories [7].

As with other forms of IRD, SMD can be associated with a variety of psychological and social problems. Several studies indicate a link between intensity of use and psychopathological symptoms, especially anxiety and depressive symptoms in particular among adolescents [9,10,11], although results are not always consistent [12]. Digital stress, triggered by predictors like FOMO (fear of missing out) or social pressure, can play a moderating role [13]. Certain personality traits are also associated with an addictive use of social media. Associations between SMD and extraversion [14], narcissism and low self-esteem [15] were reported.

Although epidemiological studies are rather rare, Müller and colleagues [16] showed that prevalence rates of problematic social media use among German adolescents amount to 4.1 percent among boys and 3.6 percent among girls, with girls using social media more frequently. Bányai and colleagues [17] identified a prevalence rate of addictive use of 4.5 percent among Hungarian adolescents. Studies that focused on the addictive use of Facebook reported prevalence rates of 1.6 percent among Nigerian adolescents [18] and 8.6 percent among Peruvian students [19]. Overall, SMD seem to mostly affect people of younger age and women [20,21,22,23,24]. 

The aim of the present study is to investigate gender-related nosological characteristics and clinical features of social media disorder (SMD). Our main research focused on the hidden prevalence of IRD in general, and specifically related to disordered social media use in female patients of different mental health care services. Secondly, we were interested in finding out more on clinical characteristics of this under-researched population, namely female patients with different subtypes of IRD by comparing these women with other female patients not meeting criteria for IRD. Since SMD is a subtype of IRD, we expected a high correlation between SMD and IRD. By comparing patients with (1) SMD, (2) other subtypes of IRD (gIRD) and (3) without IRD (comparison group) we aimed to examine gender- and IRD-specific characteristics regarding personality traits, comorbid disorder, symptom burden, and functional impairment. We also intended to differentiate the patient groups regarding to problem perception and the degree of concealment of usage behavior.

By dividing the groups and differentiating between patients with SMD and those without SMD but IRD, the social media disorder, as a subtype of IRD, should be considered explicitly in contrast to the general disorder and the other subtypes.

## 2. Materials and Methods

Data were collected within the scope of the IBSfemme-project funded by the German Federal Ministry of Health and took place from May 2018 until May 2019. Aim of the study was to identify undiagnosed comorbid INS and to examine gender-specific differences (for further information please see project report in German language [25]). The clinical consecutive survey was carried out in a total of 19 mental health care institutions, including inpatient and outpatient treatment services with different focal points of care. The final sample includes *n* = 294 women with full data available. Table 1 shows the key characteristics of the included study participants are provided.

In order to answer our research questions, we divided the total sample according to the cutoff scores of two major classification instruments. The Scale for Assessment of Internet and Computer game Addiction (AICA-S) was used to identify IRD without a further differentiation of its specific subtypes. Patients exceeding the cutoff of AICA-S were labelled as global “gIRD-group” (*n* = 30, 10.2 percent) given that they did not exceed the cutoff of the second classification instrument, Social Media Disorder Scale (SMDS). Patients exceeding the cutoff of the SMDS were labelled as “SMD-group” (*n* = 37, 12.6 percent), irrespective of their score in AICA-S. Patients not exceeding either cutoff were labelled “comparison group” (*n* = 227, 77.2 percent).

### 2.1. Measures

The main questionnaire included socio-demographics and the specific psychometric scales listed below. Additionally, an appendix was added to the questionnaire to be completed by the clinician in charge. Here, information on the main diagnosis and additional, secondary mental disorders were documented (ICD-10 encoding).

Scale for Assessment of Internet and Computer game Addiction (AICA-S) [26] is a self-report scale with 16 items (on 5-point Likert Scales). It indicates moderately addictive behavior at a cut-off of seven and severely addictive behavior at cut-off of 13.5. Frequency, duration and the intensity of use for various Internet applications (e.g., online games, shopping, social media, pornography) are also assessed. The scale has good diagnostic validity and good internal consistency (α = 0.89) [27].

Social Media Disorder Scale (SMDS) [7] is a self-report scale assessing addictive social media behavior. The short version contains nine dichotomous items, the cut-off of five classified as addictive user. Test theoretical investigations show good reliability (α = 0.82), moderate test–retest reliability as well as satisfactory convergence and construct validity.

Sheehan Disability Scale (SDS) [28] is a self-report scale that measures functional impairment in the three areas of work, social activities and family. Each area is covered by an item with a ten-point Likert scale. The cut-off of five indicates functional impairment. Good reliability was found (α = 0.89) [29].

Hopkins Symptom Checklist (HSCL-10) [30] is a self-report scale for assessing symptom severity regarding anxiety and depression. The short version includes ten items and shows good reliability (α = 0.89) [31].

Big Five Inventory (BFI-10) [32,33] is a self-report scale that measures the characteristics of the big five personality traits. The short version covers each personality trait with two items. Good reliability and validity were found (retest reliability: r = 0.75).

### 2.2. Statistical Analysis

For all data analysis, SPSS 23.0 was used. Group comparisons were examined using *t*-tests, categorical data using chi-square tests and their effect size using Cramer-V and Cohen’s d. MANCOVAS and ANCOVAS, respectively ANOVAS were additionally calculated with partial eta-squared (η^2^) as an indicator of effect size and Games Howell Test as a post hoc-measure.

## 3. Results

### 3.1. Prevalence of Internet-Related Disorders and Social Media Disorder

A total of 17.5 percent of the sample met criteria for IRD according to AICA-S and 12.5 percent of the sample met criteria for SMD. IRD was also found in 67.6 percent of women affected by SMD. Accordingly, the chi-square test indicated a significant association between the classification according to AICA-S and SMDS with a moderate effect size (χ^2^ = 66.444, *p* < 0.001, Cramer-V = 0.475).

Regarding the various internet-applications, social media or chatting are most frequently cited as the main internet activity in all three groups (SMD group: 57.1 percent, gIRD group: 31.6 percent, comparison group: 39.9 percent), while especially the SMD group uses chat programs. Streaming offers are also used often in the gIRD (26.3 percent) and the SMD group (28.6 percent) but not that frequently in the comparison group (10.8 percent). The latter instead uses information research more often (17.6 percent) or uses several areas of application with the same frequency (19.8 percent).

### 3.2. Personality Traits

The results of the group comparison with regard to the personality traits are shown in Table 2. For both, gIRD and SMD, significant negative correlations with agreeableness (SMD: r = −0.169, *p* = 0.010; gIRD: r = −0.154, *p* = 0.015) and conscientiousness (SMD: r = −0.273, *p* < 0.001; gIRD: r = −0.341, *p* < 0.001) were found. Neuroticism (gIRD: r = 0.207, *p* = 0.001) and extraversion (gIRD: r = −0.155, *p* = 0.015) correlated only significantly with gIRD while openness was only correlated with SMD (SMD: r = −0.152, *p* = 0.020). 

The results of the MANCOVA with patient-group (gIRD, SMD, comparison) as factor, age as covariate and the five personality traits according to BFI-10 as dependent variables showed a significant main effect for patient-group (F = 3.24, *p* < 0.001) and for the covariate age (*p* = 0.001). Subsequent ANCOVAS yielded significant main effects for patient-group for neuroticism, agreeableness, and conscientiousness. Only for extraversion, a significant effect of age was found (*p* = 0.002). For neuroticism the post hoc tests yielded significant group differences between gIRD and comparison (d = 0.668), gIRD and SMD (d = 0.644), for agreeableness between SMD and comparison (d = 0.514), and for conscientiousness between gIRD and comparison (d = 0.664) and SMD and comparison (d = 0.787).

### 3.3. Group Differences in Functional Impairment and Psychopathological Distress

With regard to psychosocial functioning, no group differences were found. Table 3 shows that the three groups differed only slightly. 

However, applying the recommended cutoff of 5 points for each SDS-scale, the chi-square tests displayed significant differences regarding the classification of the three groups for all three dimensions (occupational, social, family). In the occupational (31.3 percent vs. 13.0 percent vs. 11.8 percent; χ^2^ = 7.921, *p* = 0.019, Cramer-V = 0.188) and the family dimension (26.7 percent vs. 13.0 percent vs. 8.3 percent; χ^2^ = 7.097, *p* = 0.029, Cramer-V = 0.175), women of the comparison group were significantly less impaired than women in the gIRD- or SMD-group. Whereas in the social dimension significantly more women with SMD were classified as functionally impaired (73.8 percent vs. 73.9 percent vs. 94.4 percent; χ^2^ = 7.293, *p* = 0.026, Cramer-V = 0.178). 

Regarding psychopathological distress, a significant main effect was found for the score of HSCL (F(2,228) = 2.64, *p* < 0.001, η^2^ = 0.023). Yet, the post hoc test showed no significant group differences. The SMD-group (M = 26.2, SD = 4.94) and the gIRD group (M = 26.3, SD = 6.96) differed only slightly from the comparison group (M = 24.0, SD = 6.90).

### 3.4. Comorbidities

In Table 4, we report the assigned main and additional mental disorders diagnoses, which are externally assessed by clinicians. As can be seen, 58.8 percent of the total sample had a substance-related disorder, which is also the most common diagnosis, followed by mood disorders (39.1 percent), anxiety disorders (23.1 percent) and personality disorders (13.3 percent).

Group comparisons showed significant differences in the main diagnoses of substance-related disorder, schizophrenia, eating disorders, and personality disorders. Women of the gIRD group were significantly less likely to be affected by substance-use disorders (χ^2^ = 10,028, *p* = 0.007, Cramer-V = 0.185) and significantly more likely to suffer from eating disorders (χ^2^ = 6413, *p* = 0.040, Cramer-V = 0.148) than the other two groups. Both the gIRD- and SMD-group were significantly more often affected by psychotic disorders (χ^2^ = 6.975, *p* = 0.031, Cramer-V = 0.154) and personality disorders (F6x; χ^2^ = 8.865, *p* = 0.012, χ^2^ = 8.865) compared to the comparison group.

### 3.5. Relationship of Socio-Economic Data with IRD and SMD

Regarding the socio-economic characteristics (see Table 1), the gIRD and SMD groups differed from the comparison group in terms of mean age, marital status and graduation. Women without internet-related disorder were older on average (39.58 years vs. 30.73 years vs. 25.41 years; F(2,290) = 22.846, *p* < 0.001, η^2^ = 0.136) and were more often in a relationship (χ^2^ = 18.504, *p* = 0.005, Cramer-V = 0.180). Women of the gIRD group had significantly more often a high school graduation than the other two groups (χ^2^ = 22.513, *p* = 0.013, Cramer-V = 0.197). Women of the SMD group were more often unemployed than those in the comparison or gIRD group (χ^2^ = 20.022, *p* = 0.010, Cramer-V = 0.198) and lived more often with another person, but not with the partner, in one household (χ^2^ = 38,397, *p* < 0.001, Cramer-V = 0.271).

## 4. Discussion

The aim of the present study was to examine gender-related clinical characteristics of social media disorder as a subtype of internet-related disorders and to identify nosological characteristics among female patients, who were usually neglected in patient-related research. Therefore, we generated a clinical sample of *n* = 294 women, carried out by 19 mental health care institutions.

Our results indicate a higher prevalence of IRD in clinical samples compared to community-based samples and thus confirming prior study findings. This applies both to global internet-related disorder and to specific social media disorder, which at 17.5 percent and 12.5 percent are significantly higher than in the general population [34]. Regardless of any addictive use, our data demonstrate that social media use is the preferred online activity among women. The use of streaming applications is reported as the second most common with over 25 percent of the affected women, while online games are relatively rarely reported as the main activity (less than 10 percent). 

As expected, there is a substantial correlation between gIRD and SMD. This is reasonable since gIRD represents the global construct or “umbrella term” where SMD can be identified as a more specific subtype [35,36]. Yet, at the same time, a third of women with social media disorder do not exceed the cutoff for the assessment of global IRD. This seems particularly relevant since especially women are still underrepresented in research and treatment [20,37]. An explanation for the imbalance of men and women in treatment and research might therefore be that women affected by the global measuring instruments are more often false negative. On the one hand, this can be a problem of gender-specific diagnosis of IRD, on the other hand, relevant features of the SMD subtype may not have been sufficiently taken into account in the global measuring instrument.

The main subject of the study was the identification of nosological characteristics in SMD with particular attention to personality traits, comorbidities, psychopathological distress, and functional impairment.

The Big Five personality traits have been investigated frequently in relation with IRD [38]. Generally, neuroticism was positively, conscientiousness, openness, extraversion and agreeableness are negatively related to IRD, whereby conscientiousness has the greatest effect size. Yet, most of the available studies are focusing on either IRD as a global construct or Gaming Disorder as a specific subtype. Additionally, systematic gender-specific differences in these traits have been investigated to a lesser extent, but so far there is still a predominance of men in previous studies. Andreassen and colleagues [39] examined personality traits in relation to different behavioral addictions. With regard to addictive social media use (Facebook), they found a negative correlation with extraversion, openness and conscientiousness. In contrast, no associations with neuroticism were found for SMD. Similar relationships were found in this study: While the group comparisons indicated lower levels of conscientiousness in both internet-related groups (gIRD and SMD), significantly increased neuroticism was only found in the global gIRD-group. No group differences were found for openness and extraversion that showed only small correlations. However, the SMD-group differs significantly from the comparison group in terms of agreeableness, which is less pronounced in the SMD-group. Agreeableness is generally related to interpersonal behavior. Low agreeableness is characterized as being less socially adapted, more suspicious, unrelenting, and rather competitive than cooperative. Previous research results have shown that usage motives of women are socially oriented and serve to intensify existing social contacts, whereby emotional stimuli and family relationships are the focus of self-representation [22,40]. Low levels of agreeableness in our sample appear to contradict these previous findings at first glance. Yet, we also found that female patients with SMD revealed significant higher impairment in social functioning than comparisons and female patients with global gIRD. This is a relevant finding potentially uncovering a specific nosological feature of SMD. 

An explanation for these findings might be found in the study by Andreassen and colleagues [15] who reported a relationship between SMD and narcissism. The authors suspect that online platforms fulfill the need for belonging and confirmation of the idealized self. This is supported by the results of previous studies, in which the connection between narcissism, functional disorders, and interpersonal strain [41] as well as low levels of agreeableness and conscientiousness [42] were documented. Additionally, the heightened rates of comorbid personality disorders might be explainable by these findings. A clinical characteristic of borderline personality disorder regards severe problems in social relationships and social interactions [43]. One could speculate that—for these patients—virtual contacts and online conversations are less prone to conflicts because of the non-direct, non-confrontational type of interaction and social feedback which could explain the preference for virtual communication. However, there are no significant differences between the SMD- and gIRD-group with regard to personality disorders. In both groups, personality disorders and schizophrenic disorders occur significantly more frequently than in the comparison group and can therefore be regarded as being related to the global IRD phenomenon. In contrast, in comparison with gIRD-patients, patients with SMD are suffering more often from substance-related disorders and less from eating disorders. Therefore, it can be carefully concluded that IRD (global and specific) in women is significantly associated with severe mental disorders.

With regard to patient characteristics, we found that especially younger women are affected by internet-related disorders, both global IRD and specific SMD). The average age of 30 years of the gIRD-group is nine years below the average age of the comparison group; women of the SMD-group (at average 25 years) are five years younger than comparison subjects. Further they are more often single and less likely to have a school graduation or are more still attending school. Whereas women in the gIRD-group have more often a high school diploma and differ significantly in this respect from both other groups. It is also striking that, compared to the gIRD-group, the women in the SMD group are more often unemployed and more often live in a common household with other people, but not with their partner. This could be justified by younger age.

Summarizing, the results indicate that patients who have an internet-related disorder (both global and specific) have similar and common characteristics in comparison to the comparison group on the one hand. On the other hand, there are nosological characteristics in women with social media disorder compared to those meeting criteria for IRD in general. The disorder appears to have one thing in common: compared to the comparison group, the affected women are younger, are significantly more frequently functionally impaired, display lower scores in conscientiousness, reveal more often comorbid disorders regarding schizophrenia and personality disorders. 

Compared to patients who meet diagnostic criteria for IRD but regarding social media, patients with SMD are less pronounced in the personality traits neuroticism and agreeableness, more often functionally impaired in the social dimension, meet more often criteria of substance-use disorders, while being and less often affected by eating disorders. They also are more often unemployed and live more often with people other than their own partner. They were also on average five years younger than the IRD—patients, although this difference was not significant.

The study has some limitations. As the sample is solely female, the results cannot be transferred to males. Previous gender comparative studies in the field of internet-related disorders have shown differences both in terms of use and motivation as well as in clinical characteristics [22,44]. Since women in particular are affected by excessive social media use, the sample should remain focused on this gender in this study, in further studies male participants can also be included. Another limitation concerns group formation. Since there is a significant correlation between SMD and IRD, the two groups are not fully distinctly formed. Within the SMD group, 67 percent of the subjects also scored in AICA-S. Since this was to be expected and in particular the social media disorder is at the center of this study, this is not a problem in terms of test theory. Furthermore, a comparison with a comparison group that does not originate from the clinical setting would be very interesting, since it would be expected that there would be further differences in the examined measurement parameters. In this case, the clinical sample is interesting in that a clinical prevalence of IRD and SMD can be measured.

## 5. Conclusions

The results of the present study indicate that nosological characteristics can also be found in women with social media disorder in comparison to women with global internet-related disorders. Particularly noteworthy here are a lower level of agreeableness in and increased social functional impairment by women with SMD. From this it can be deduced that the subtypes of internet-related disorders differ in their characteristics and specifications. In view of the steadily increasing use of social media and social networking sites, it seems important to continue to examine the disorder of internet-related use in detail.

## Figures and Tables

**Table 1:** Sample characteristics.

	Women Total	SMD-Group	gIRD-Group	Comparison-Group
N	294	37	30	227
age (M, SD)	36.88 (13.87)	25.41 (7.69)	30.73 (12.96)	39.58 (13.59)
marital status (%, N)				
unmarried, single	35.6 (101)	61.8 (21)	46.7 (14)	30.0 (66)
unmarried, partnership	21.1 (60)	23.5 (8)	20.0 (6)	20.9 (46)
married	20.4 (58)	5.9 (2)	16.7 (5)	23.2 (51)
separated	22.9 (65)	8.8 (3)	16.7 (5)	25.9 (57)
housing situation (%, N)				
alone	32.8 (86)	11.1 (4)	24.1 (7)	38.1 (75)
with partner	27.5 (72)	5.6 (2)	24.1 (7)	32.0 (63)
with other	39.7 (104)	83.3 (30)	51.7 (15)	29.9 (59)
graduation (%, N)				
without	3.5 (10)	11.1 (4)	6.7 (2)	1.8 (4)
still in school	5.5 (16)	11.1 (4)	6.7 (2)	4.5 (10)
lower secondary school	23.2 (67)	25.0 (9)	10.0 (3)	24.7 (55)
secorndary school	38.1 (110)	36.1 (13)	26.7 (8)	39.9 (89)
high school	25.6 (74)	11.1(4)	43.3 (13)	25.6 (57)
other	4.2 (12)	5.6 (2)	6.7 (2)	3.6 (8)
occupational status (%, N)				
employed	38.7 (99)	22.2 (6)	37.5 (9)	41.0 (84)
housewife/-man	6.3 (16)	0 (0)	8.3 (2)	6.8 (14)
student	10.5 (27)	7.4 (2)	25.0 (6)	9.3 (19)
unemployed	38.7 (99)	70.4 (19)	25.0 (6)	36.1 (74)
retired	5.9 (15)	0 (0)	4.2 (1)	6.8 (14)
migration background (%, N)				
yes	10.8 (31)	8.6 (3)	10.7 (3)	11.2 (25)

Notes: M = Mean; SD = standard deviation; gird = global internet-related disorder, classified by AICA-S; SMD = Social Media Disorder, classified by SMDS.

**Table 2:** Personality traits.

	Women Total	SMD-Group	gIRD-Group	Comparison-Group	Statistics
Neuroticism					F(2,228) = 4.72
(M, SD)	3.71 (0.903)	3.73 (0.865) ^B^	4.26 (0.619) ^A^	3.67 (0.890) ^B^	*p* = 0.010
					η^2^ = 0.040
Extraversion					
(M, SD)	2.94 (1.004)	2.83 (0.792)	2.65 (1.049)	3.06 (1.037)	n.s.
openness					
(M, SD)	3.45 (1.045)	3.13 (0.982)	3.39 (1.065)	3.54 (1.073)	n.s.
agreeableness					F(2,228) = 4.36
(M, SD)	3.31 (0.912)	2.90 (0.868) ^A^	3.06 (1.141)	3.35 (0.856) ^B^	*p* = 0.014
					η^2^ = 0.037
conscientiousness					F(2,228) = 13.09
(M, SD)	3.75 (0.825)	3.23 (0.769) ^A^	3.34 (0.789) ^A^	3.88 (0.799) ^B^	*p* < 0.001
					η^2^ = 0.103

Notes: M = Mean; SD = standard deviation; IRD = global internet-related disorder; SMD = Social Media Disorder; F = F value (main effect MANOVA); η^2^ = eta square; *p* = *p* value; superscripts show significant group differences (post hoc: Games-Howell-test); n.s. = not significant (*p* > 0.05).

**Table 3:** Functional impairment level.

Functional Impairment Level	Women Total	SMD-Group	gIRD-Group	Comparison-Group	Statistics
SDS occupational					
score (M, SD)	6.26 (3.093)	6.76 (2.230)	7.09 (2.937)	6.20 (3.159)	n.s.
not impaired (%)	28.1	11.8	13.0	31.3	*p* = 0.019
impaired (%)	71.9	88.2	87.0	68.7	Cramer-V = 0.188 χ^2^ = 7.921
SDS social					
score (M, SD)	6.25 (2.867)	7.17 (1.732)	6.13 (2.881)	6.17 (2.983)	n.s.
not impaired (%)	24.1	5.6	26.1	26.2	*p* = 0.026
impaired (%)	75.9	94.4	73.9	73.8	Cramer-V = 0.178 χ^2^ = 7.293
SDS family					
score (M, SD)	6.28 (2.818)	7.11 (1.894)	6.52 (2.410)	6.09 (3.017)	n.s.
not impaired (%)	22.2	8.3	13.0	26.7	*p* = 0.029
impaired (%)	77.8	91.7	87.0	73.3	Cramer-V = 0.175 χ^2^ = 7.097

Notes: M = Mean; SD = standard deviation; IRD = global internet-related disorder; SMD = Social Media Disorder; χ^2^=chi square; *p* = *p* value; Cramer-V = effect size indicator; n.s. = not significant (*p* > 0.05).

**Table 4:** Assigned main and additional mental disorders diagnoses, externally assessed by clinicians.

	Women Total	SMD-Group	gIRD-Group	Comparison-Group	Statistics
F1x (%) Mental and behavioural disorders due to psychoactive substance use	58.8 (173)	54.1 (20)	33.3 (10)	63.0 (143)	*p* = 0.007 Cramer-V = 0.185 χ^2^ = 10.028
F2x (%) Schizophrenia, schizotypal and delusional disorders	6.5 (19)	13.5 (5)	13.3 (4)	4.4 (10)	*p* = 0.031 Cramer-V = 0.154 χ^2^ = 6.975
F3x (%) Mood [affective] disorders	39.1 (115)	32.4 (12)	56.7 (17)	37.9 (86)	n.s.
F4x (%) Neurotic, stress-related and somatoform disorders	23.1 (68)	16.2 (6)	23.3 (7)	24.2 (55)	n.s.
F5x (%) Behavioural syndromes associated with physiological disturbances and physical factors	10.5 (31)	5.4 (2)	23.3 (7)	9.7 (22)	*p* = 0.040 Cramer-V = 0.148 χ^2^ = 6.413
F6x (%) Disorders of adult personality and behaviour *	13.3 (39)	21.6 (8)	26.7 (8)	10.1 (23)	*p* = 0.012 Cramer-V = 0.174 χ^2^ = 8.865
F9x (%) Behavioural and emotional disorders with onset usually occurring in childhood and adolescence	5.1 (15)	10.8 (4)	6.7 (2)	4.0 (9)	n.s.

Notes: % = percent value; IRD = global internet-related disorder; SMD = Social Media Disorder; Cramer-V = effect size indicator; χ^2^ = chi square; *p* = *p* value (level of significance); n.s. = not significant (*p* > 0.05); * among that ICD-chapter, only personality disorders were included.

## Data Availability

The data presented in this study are available on request from the corresponding author. The data are not publicly available due to reason of data protection.
